# Supplementary material for: Learning curve and surgical outcome of robotic assisted colorectal surgery with ERAS program
Source: Sci Rep. 2022 Nov 29;12:20566. doi: 10.1038/s41598-022-24665-w (PMC9709162; doi:10.1038/s41598-022-24665-w)
Supplement: Supplementary file 1 — Supplementary Information 1. [file 41598_2022_24665_MOESM1_ESM.zip › LSC Raw data-Ñ[▒K/123/N_ñw╝╨░O▒Kñσ.pdf]

W95 / W95

臺中榮民總醫院

手術室護理記錄

[redacted]

基本資料

- 室別： 二醫手術室-31室，手術科別： CRS ，手術類別： 常規，日期： 2021/03/24
- 進入時間： 1015 ； 麻醉開始時間： 1020 ； Time Out時間： 1120 ； 劃刀時間： 1121 ； 手術結束時間： 1600 送出時間： 1615 ；
- 麻醉方式： GE

  診斷： S COLON CA S/P LOOP

  手術名稱： Low anterior resection +/- pelvic LN dissection
  Ileostomy
  Laparoscopy

  手術局部用藥： nil

  術前預防性抗生素： 無
- 手術醫師： 林俊余，林陳彥，楊立宇
- 麻醉醫師： VS 蔡志忠
-
  - 刷手護士A ： 李庭，時間： 1015，接替刷手一： 吳雅華，時間： 1230，接替刷手二： 王毓均，時間： 1500
  - 流動護士A ： 吳雅華，時間： 1015，接替流動一： 王心俞，時間： 1200，接替流動二： 李庭，時間： 1300

皮膚狀況

A-V Shunt： 無

術前： 不完整

- 1.紅腫

部位(術前)：

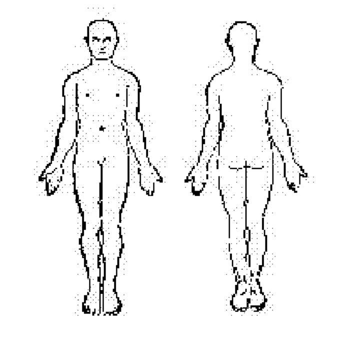

左手上臂破皮貼人工皮，前臂紅腫，全身皮膚乾燥斑駁顏色

記錄者： 上次簽章: 吳雅華

術後： 不完整

- 1.紅腫

部位(術後)：

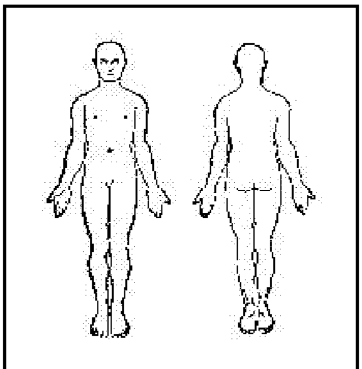

左手上臂破皮貼人工皮，前臂紅腫，全身皮膚乾燥斑駁顏色

記錄者： 李庭

手術準備

手術臥式 單一手術臥式

-
  - 手術臥式： 截石臥式
  - 使用附件： 矽膠枕頭，腳架
  - 皮膚準備： 10%Aq Betadine，Hibiscrub，2% Chlorhexidine
  - 電燒： 使用電燒: 電極片位置： 其它： 迴路電極墊，記錄者： 上次簽章: 吳雅華

止血帶T： 無

植入物

- 植入物名稱/號碼/數量/部位： Hyalobarrier gel 10ml/壹/腹腔

, 記錄者： 上次簽章: 李庭

標本送檢

- 細菌培養 無
- 細胞學 無
- 冰凍切片 無
- 病理標本
  組織名稱一： distal cutend
  ,
  組織名稱二： IMA ROOT
  ,
  組織名稱三： R-S colon
- 截肢 無

標本交付 標本： 貳 袋，標本： 零 盒，標本： 壹 瓶，細胞學： 零 管，Culture Tube： 零 管，被交付醫師： CR 林陳彥，

- 記錄者： 上次簽章: 李庭
- 時間： 1541

傷口術後敷料

傷口敷料：

- 請於圖上標示部位：

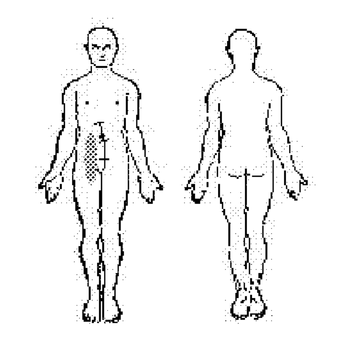

直切傷口及腹腔鏡切口

- 傷口敷料： 白紗及mepore

導管

導管	天數	導管資訊	插入日期	插入時間	更換日期	拔除日期	拔除時間	拔除原因	預計拔除/更換	預計拔除/更換日期
Foley(1)	1	號碼:16 Fr.; 部位:膀胱;	2021/03/24	1040						
Penrose Drain(1)	1	號碼:12 mm.; 部位:骨盆腔;	2021/03/24	1450						
Sump NG Tube(1)	1	號碼:16 Fr.; 部位:胃; 註記:fix 55cm;	2021/03/24	1055						
A-line (1)	1	號碼:20 G; 部位:前臂; 象限:RU;	2021/03/24	1035						

用物計數

- 手術中出血量： 250 c.c.
- 手術中尿量： 450 c.c.
-
    - 其它： 無

用物計數： 多切口同時縫合

- 1.器械： 正確，2.縫針： 正確，3.刀片： 正確，4.紗布： 正確，5.腹部墊： 正確，6.紗球： 未使用，7.PEANUTS ： 未使用，8.紗條： 未使用，9.絲帶： 未使用，10.針頭： 未使用，11.Vessel loop： 未使用，12.Boots： 未使用，13.Jelco： 未使用，14.棉條： 未使用，
    - 第一次：
        - 刷手護士 ： 吳雅華
        - 流動護士： 上次簽章: 李庭
        - 時間： 1500

  ,
    - 第二次：
        - 刷手護士 ： 王毓均
        - 流動護士： 上次簽章: 李庭
        - 時間： 1520

  ,
    - 第三次：
        - 刷手護士 ： 王毓均
        - 流動護士： 上次簽章: 李庭
        - 時間： 1540

備註

備註： NIL

- 記錄者： 李庭
- 時間： 1700

填寫護理人員 李庭

正式摘要

W95 / W95

doctorRank:B
